# Mobilizing social support networks to improve cancer screening: the COACH randomized controlled trial study design

**DOI:** 10.1186/s12885-015-1920-7

**Published:** 2015-11-16

**Authors:** Olive Mbah, Jean G. Ford, Miaozhen Qiu, Jennifer Wenzel, Lee Bone, Janice Bowie, Ahmed Elmi, Jimmie L. Slade, Michele Towson, Adrian S. Dobs

**Affiliations:** Department of Oncology, Johns Hopkins School of Medicine, Baltimore, MD USA; Department of Medicine, Einstein Healthcare Network, Philadelphia, PA USA; Medical Oncology Department, Cancer Center of Sun Yat-sen University, Guangzhou, China; Department of Acute and Chronic Care, Johns Hopkins School of Nursing, Baltimore, MD USA; Department of Health, Behavior and Society, Johns Hopkins Bloomberg School of Public Health, Baltimore, MD USA; Community Ministry of Prince George’s County, Upper Marlboro, MD USA; Maxwell Enterprises, Baltimore, MD USA; Department of Medicine, Johns Hopkins School of Medicine, Baltimore, MD USA

**Keywords:** Cancer screening, African Americans, Older adults, Family patient navigators, CBPR

## Abstract

**Background:**

Disadvantaged populations face many barriers to cancer care, including limited support in navigating through the complexities of the healthcare system. Family members play an integral role in caring for patients and provide valuable care coordination; however, the effect of family navigators on adherence to cancer screening has not previously been evaluated. Training and evaluating trusted family members and other support persons may improve cancer outcomes for vulnerable patients.

**Methods:**

Guided by principles of community based participatory research (CBPR), “Evaluating Coaches of Older Adults for Cancer Care and Healthy Behaviors (COACH)” is a community-based randomized controlled trial to assess the effectiveness of a trained participant-designated coach (support person or care giver) in navigating cancer-screening for older African American adults, 50–74 years old. Participants are randomly assigned as dyads (participant + coach pair) to receiving either printed educational materials only (PEM—control group) or educational materials plus coach training (COACH—intervention group). We defined a coach as family member, friend, or other lay support person designated by the older adult. The coach training is designed as a one-time, 35- to 40-minute training consisting of: 1) a didactic session that covers the role of the coach, basic facts about colorectal, breast and cervical cancers (including risk factors, signs and symptoms and screening modalities), engaging the healthcare provider in cancer screening, insurance coverage for screening, and related healthcare issues, 2) three video skits addressing misconceptions about and planning for cancer screening, and 3) an interactive role-play session with the trainer to reinforce and practice strategies for encouraging the participant to get screened. The primary study outcome is the difference in the proportion of participants completing at least one of the recommended screenings (for breast, cervix or colorectal cancer) between the control and intervention groups.

**Discussion:**

Building on trusted patient contacts to encourage cancer screening, COACH is a highly sustainable intervention in a high-risk population. It has the potential to minimize the effect of mistrust of the medical establishment on screening behaviors by mobilizing participants’ existing support networks. If effective, the intervention could have a high impact on health care disparities research across multiple diseases.

**Trial registration:**

ClinicalTrials.gov (NCT01613430). Registered June 5, 2012

## Background

Cancer is the second leading cause of death in the United States, and accounts for one in every four deaths [1]. Advances in early diagnosis and treatment have led to declining mortality rates over the past two decades, but racial/ethnic disparities persist [2–5]. By 2030, cancer incidence is expected to increase by 99 % among minorities (vs. 31 % for whites) [6]. Compared to other racial/ethnic groups, African Americans have the highest death rates and shortest survival for most cancers [1, 7]. When stratified by age and race, older African Americans bear the highest mortality burden [8]. Reasons for these ongoing disparities are multifactorial but inadequate secondary prevention through screening and late diagnosis have been shown to play an important role [7, 9–14], highlighting the need for effective and sustainable interventions.

Among the many unique barriers that minority populations face in cancer care are difficulties navigating through the complexities of the healthcare system [15]. The use of care coordination models, including community health workers (CHW) or patient navigators (PN), have demonstrated positive outcomes. These include bridging health care systems and racial/ethnic minority communities in various settings, including for cancer screening [15–19] and diagnostic follow up [18, 20–23]. However, prior patient data revealed that trusted social network members play an even more integral role in caring for patients and may be the most effective at helping to coordinate care and address their cancer-related needs [24, 25]. As care has shifted from the hospital to the home, caregivers have been tasked with complex, multifaceted responsibilities that many are ill prepared to assume [24]. Furthermore, our work with African American older adults suggest that 1) this population considers family and friends as trusted sources of cancer information and 2) both urban and rural African American cancer survivors preferred to work through healthcare access issues with people with whom they already had a relationship (vs. CHWs whom they did not know and who did not have formal health education training) [25, 26]. Additionally, older adult patients are frequently accompanied by family members or other support persons to medical appointments; companions who are verbally active in these visits are generally effective in improving patient-physician communication [27]. As the reliance on family caregivers increases, of noted importance for minority patients, programs to build caregiver confidence and competence provide opportunities to address existing gaps within the healthcare system.

Although a growing body of literature shows the benefits of caregiver-related interventions, there’s limited evidence on their effects on outcomes in cancer care, other than for depression and psychological functioning [28, 29]. To our knowledge, there are no published caregiver-related interventions targeting cancer screening. There is a need for rigorous evidence-based research describing and evaluating interventions where caregivers assist in navigating older adults. The “Evaluating Coaches of Older Adults for Cancer Care and Healthy Behaviors (COACH)” study also addresses the essential need to evaluate the effects of such interventions on caregiver outcomes [30]. The central hypothesis of the COACH study is that members of older adults’ extended families or support networks can be trained to be effective coaches, who provide support through the cancer control continuum (i.e., prevention, screening, diagnosis and treatment). The primary outcome of the study is adherence to cancer screening.

## Methods

### Study design, population and setting

**Fig. 1:**
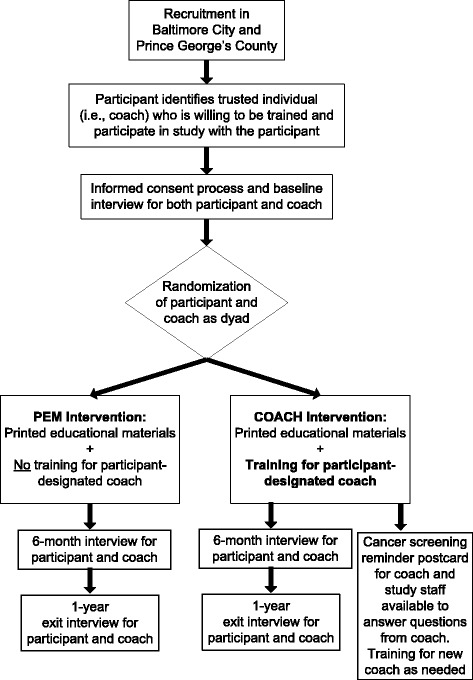
Evaluating Coaches of Older Adults for Cancer Care and Healthy Behaviors (COACH) Study Design Flow Chart

The COACH study is an ongoing community-based randomized controlled trial to assess the effectiveness of trained participant-designated coaches vs. the more standard intervention of distributing printed educational materials in improving cancer-screening behaviors. Participants are randomly assigned as dyads (participant + coach pair) to receiving either printed educational materials only (PEM—control group) or educational materials plus coach training (COACH—intervention group) Fig. 1. The study population consists of African American older adults 50 to 74 years old who reside in Baltimore City or Prince George’s County, Maryland. The populations of both Baltimore City and Prince George’s County are approximately 63 and 65 % African American respectively. Baltimore City’s overall socioeconomic status is lower than that of Prince George’s County as indicated by median household income ($41,385 vs. $73,623) and percent of persons living below the poverty line (23.8 vs. 9.4 %) [31, 32]. Nevertheless, compared to the overall population of the U.S. and of the State of Maryland, both counties show higher age-adjusted cancer mortality rates for breast, cervix and colorectal cancer [33]. The similarities in cancer risk and mortality profiles between these two counties offer a potentially rewarding context for: 1) disaggregating the effects of race/ethnicity vs. socioeconomic status as determinants of adverse health outcomes; and 2) evaluating how the effects of interventions to reduce cancer disparities may vary by socioeconomic status. Other inclusion criteria for the study are: eligibility for breast, cervical or colorectal cancer screening at time of enrollment, and having a support person (coach) who is available to participate in the study. Coaches can be a family member, friend, neighbor or other lay support person designated by the participant. Exclusion criteria include a diagnosis of cancer within the past 5 years or a diagnosis of cancer not in remission, an inability to provide informed consent, or current residence in a chronic care facility or otherwise institutionalized. The rationale for excluding individuals who are institutionalized is that they may already be receiving specific interventions or face special barriers that affect the outcome variables of interest. To preserve the integrity of the trial, only one individual per household is eligible to participate. Randomization is stratified by county and gender.

### Recruitment

Participants were recruited into the study between June 2012 and July 2015 using both convenience- and a quasi-population- based sampling. Recruitment began with a cohort of participants from a previous cancer screening study (the Cancer Prevention and Treatment Demonstration (CPTD) Screening Trial control group participants, described in detail here— [19, 34]) who had agreed to be contacted for future studies and were within the age group for the COACH study. These individuals were contacted by phone to determine their interest in and eligibility for the COACH study. Other convenience sampling was conducted in collaboration with our community partners and consisted of targeted outreach events at community centers, churches, senior residential buildings, health fairs, and other events and locations that included our target population. Potential participants were introduced to the study and given the opportunity to be screened for eligibility. The quasi-population-based sampling entailed using commercially obtained rosters of our target population for study recruitment. This data was purchased from Aristotle International, a private company that specializes in compiling and managing voter and consumer information. The data included names and contact information for African American residents of Baltimore City and Prince George’s County, Maryland who were 50–74 years old. These potential participants were initially contacted by letter to introduce the study and provided with an opportunity to opt out of being called for recruitment by study staff. Individuals who did not opt out within a week were then contacted by phone to determine their interest in and eligibility for the study. All potential participants were screened for eligibility by phone (predominantly) or in person. Eligible individuals were consented in person, administered the baseline interview and then randomized 1:1 to either the PEM or COACH group. Participants are enrolled in the study for one year and are administered a 6-month and 1-year follow up interviews. The COACH study was approved by the Johns Hopkins School of Public Health’s Institutional Review Board.

### Interventions

#### Printed educational materials (PEM)

Participants randomized to the control group receive educational brochures on colorectal [35], breast [36] and cervical cancer [37] screenings produced by the National Cancer Institute (breast), Centers for Medicare and Medicaid Services (cervical) and the Centers for Disease Control and Prevention (colorectal).

#### Coach training

Intervention group participants receive the same PEM plus the training of their coaches, a cancer screening reminder postcard 6 weeks after enrollment into the study, and the opportunity to call study staff with any questions related to screening. The coach training is a one-time, in-person, 35- to 40-min training administered to the coach at the time of participant enrollment. Training includes: 1) a didactic session that covers the role of the coach, basic information on colorectal, breast and cervical cancers (including risk factors, signs and symptoms and screening modalities and frequency), how to prepare for a medical visit, discussing cancer screening with a healthcare provider, insurance coverage and no cost screening resources, and related healthcare issues, 2) three video skits (4 min total) addressing misconceptions around cancer screening and working with a healthcare provider and support person to plan for screening, and 3) an interactive role-play session with the trainer to emphasize strategies for encouraging the participant to get screened. The video skits and role-play help reinforce and practice strategies for encouraging the participant to get screened. Coaches are administered a pre- and post- assessment to evaluate change in knowledge and understanding of the material. As part of the training, coaches are given a packet that includes: a copy of the didactic presentation, cancer screening brochures, a points-to-remember sheet to document cancer screening questions for the participant’s healthcare provider and provider recommendations, a suggested task list for the coach, and resources that address well-known barriers to screening such as insurance coverage and transportation. Participants whose coaches disenroll from the study are given an opportunity to have a new support person trained as a coach. To standardize the coach training, all study staff who administer the training are provided with detailed guiding scripts that cover all aspects of the training. Intervention coaches are also provided with information to contact study staff with screening-related questions. Cancer screening guidelines in the coach training are based on recommendations from the U.S. Preventive Services Task Force [38] and the American Cancer Society [39]. These recommendations include: colorectal screening in adults beginning at age 50 (frequency depends on modality), breast cancer screening beginning at age 40 every 1–2 years, and cervical cancer screening beginning at 21 every 3–5 years (with a recommendation for women 65 and over to consult with their doctors). Given the expected diversity in cancer risk among study participants and differences in screening recommendations, the intervention was designed to address individual provider-driven recommendations for cancer screenings (i.e., coaches are encouraged and provided with tools to discuss cancer screening with the participant's healthcare provider).

### Integration of community based participatory research principles

The COACH study is anchored in the principles of Community Based Participatory Research (CBPR) [40, 41]. The study was designed with the active participation of community partners, including representatives of major organizations and health care institutions as well as community members from Baltimore City and Prince George’s County. Participation of community partners occurred at the level of: 1) problem definition and conceptualization; 2) collaborative review of preliminary data; 3) formulation of study hypotheses; 4) development of draft proposal for the study; and, 5) formation of two Community Advisory Groups (one in each study jurisdiction) to oversee the study. The formative phase of the study included key informant in-depth interviews with 12 health practitioners and community leaders within the two counties. Interviews explored the feasibility of the study design, barriers to screening, issues for training participant-designated coaches and providing feedback on the baseline questionnaires. The two broadly representative Community Advisory Groups (CAGs) continue to help guide all phases of the study, including review of study documents. Community Advisory Group members include representatives from community associations, faith-based organizations, local health departments and senior housing facilities, non-profit organizations, local hospitals and clinics, as well as community members.

### Data collection and study measures

All participants (including coaches) are administered a baseline, 6-month and 1-year exit interview. The baseline interview is conducted in person, the 6-month by phone and the exit interview either in-person or by phone. In-person interviews are conducted primarily at participants’ homes or study site located at a community health center. The primary focus of the interview questionnaires is to ascertain information on intermediary and outcome variables for the study, including measures for predisposing, reinforcing, and enabling factors for cancer screening. Under predisposing factors, measures of knowledge, attitudes, beliefs about cancer screening, physical and emotional functional health, cancer risk and past screening behaviors are collected. Re-enforcing factors include measures of trust in the health care system and perceived discrimination—both of which can be powerful predictors of adherence to treatment recommendations [42, 43], satisfaction with care, available support to navigate the health system and perceived self-efficacy to accomplish cancer screening. Enabling factors include items to assess the perceived ease of accessing health care services, the availability and cost associated with care, the perceived ease of scheduling health care appointments, and a standard checklist of co-morbid conditions. Other variables collected include health literacy, cognitive functioning, perceived stress and sociodemographic characteristics (age, gender, ethnicity, race, education, income, marital status and housing situation). Adherence to screening is documented through self-report of cancer screening behaviors; participants’ consent to obtain medical records and Medicare claims data is also obtained. Questionnaire items are based on published literature and uses standard instruments and scales when possible.

### Outcome variables

The primary research question to be addressed by this study is whether the COACH intervention will improve cancer screening adherence compared to the standard (control) PEM intervention. This primary question will be assessed by the difference in the proportion of participants adhering to at least one of the recommended screenings, i.e. mammogram, Pap smear, or colorectal screening. The differences in the cancer screening rates will be examined with both multivariate regression methods and survival analysis, specifically to adjust for withdrawal and right censoring over the study period. Participants who are lost to follow up will be censored at their last visit or interview for the study. Missing data mechanisms (e.g., missing at random (MAR), missing completely at random (MCAR), and non-ignorable missingness (MNAR)) will be evaluated. The distribution of “missingness” will also be assessed by comparing demographic characteristics and baseline outcome variables between participants who completed study questionnaires to those lost to follow up for each intervention arm. To account for missing data, we will consider inverse weighted probability for complete case missing data and multiple imputation methods for item missing data. The analytic strategy will also include methods for handling correlation of repeated measures, such as the Generalized Estimating Equations (GEE) method. All analyses will be carried out according to intention to treat (ITT). Given the study’s focus on provider-driven recommendations for cancer screenings, we included the following additional primary outcome posterior: the difference in the proportion of participants who report talking with their healthcare provider regarding at least one of the recommended cancer screening(s) between the control and intervention groups. Similar statistical methods will be applied here as in the analysis for screening adherence. All statistical models will be adjusted for potential confounders based *a priori* on scientific literature, or exploratory analysis such as bivariate screening.

Secondary outcome variables will include between-group differences in the time to completion of screenings, completion of other preventive screenings, changes in cancer screening barriers, and changes in the reported levels of stress for both the participants and coaches. Several outcomes will also be assessed among the coaches including differences in cancer screening adherence between coaches in the intervention and control groups, predictors of screening at follow up, satisfaction with the intervention, barriers to screening and other outcomes.

#### Power calculation

The target sample size for the study is 550 participants and 550 coaches (total *N* = 1100 participants). This is the minimum sample size required to show a 13.6 % increase relative to a 35 % anticipated screening proportion among the PEM group during follow up (based on calculations in a similar population from our previous study, the Cancer Prevention and Treatment Demonstration) with a 0.05 two-sided significance level, 20 % Type II error (80 % power), and then taking into account an anticipated loss to follow up of 20 %. Power calculations were performed using STATA/SE 11.2 (Stata Corporation, College Station, TX, USA).

## Discussion

Cancer disparities are known to result from inequities in the delivery of and access to high-quality cancer prevention, early detection, and treatment services [1]. Racial and ethnic minorities are disproportionately represented among individuals of low socio-economic status (SES), and there is a strong association between SES and reduced screening rates for all cancers [44, 45], later-stage diagnosis and poorer survival rates [46–49]. In addition to socio-economic barriers, any programs to reduce racial disparities in cancer care among older adults must mitigate other potential barriers such as management of multiple comorbidities, limited health literacy and cognitive impairment which have been associated with adverse cancer prognosis [50–56]. Given the persistence of cancer disparities, there’s a need for novel, simple, sustainable and scalable interventions targeted towards helping minorities navigate the healthcare system around cancer-related care and that also incorporate patient-preferred support systems. Building on individual and collective strengths and resources, such as those available through patients’ communities social networks, is essential to addressing disparities affecting racial/ethnic minority populations. Care models such as the use of patient navigators and CHWs show great promise; however, they have been adopted only on a limited basis, especially around cancer prevention. Specific concerns regarding their sustainability include personnel costs, as well as the variability and variable efficacy of these models [18].

Family-involved interventions in cancer care have shown promise in improving outcomes for depression/anxiety, general psychological functioning, relationship adjustment and patient symptoms such as pain [28]. Griffin et al. highlight the limited progress made in the U.S. through family-delivered interventions despite the important role families already demonstrate in cancer patients’ care [28, 29]. Yet few or no studies have reported on the patient-related cancer outcome of health care utilization [28]. There is a need to test the effect of family- or social network-based interventions on a broad scope of outcomes, including those related to improving healthcare utilization among minority cancer patients.

Our central hypothesis centers on the capacity of a trained participant-designated caregiver or support person to deliver an effective intervention to improve coordination of services related to cancer screening and related comorbidities in a cost-effective manner, a question which has received scant attention. This is a logical next step in the natural evolution of caregiver research and cancer screening interventions. The COACH model is significant because it evaluates a highly replicable and sustainable intervention in a high-risk population. It is innovative because it minimizes the effect of mistrust of the medical establishment on screening behaviors by mobilizing participants’ social support networks. If participant-designated coaches are effective in reducing disparities in cancer screening, this will provide further evidence for the potential for this low-cost intervention to improve health-related outcomes for patients and caregivers, across multiple diseases and conditions.

## Abbreviations

CAG: Community Advisory Group
CBPR: Community Based Participatory Research
CHW: Community Health Worker
COACH: Evaluating Coaches of Older Adults for Cancer Care and Healthy Behaviors
CPTD: Cancer Prevention and Treatment Demonstration
GEE: Generalized Estimating Equations
ITT: Intention to Treat
MAR: Missing At Random
MCAR: Missing Completely At Random
MNAR: Missing Not At Random
PEM: Printed Educational Materials
PN: Patient Navigator
SES: Socio-economic Status
